# System level characterization of small molecule drugs and their affected long noncoding RNAs

**DOI:** 10.18632/aging.102581

**Published:** 2019-12-18

**Authors:** Haixiu Yang, Yanan Jiang, Yunpeng Zhang, Yanjun Xu, Chunlong Zhang, Junwei Han, Fei Su, Xiaoqi Liu, Kai Mi, Bing Liu, Desi Shang

**Affiliations:** 1 College of Bioinformatics Science and Technology, Harbin Medical University, Harbin 150081, China; 2 Translational Medicine Research and Cooperation Center of Northern China, Heilongjiang Academy of Medical Sciences, Harbin 150081, Heilongjiang, China; 3 Department of Pharmacology (State-Province Key Laboratories of Biomedicine-Pharmaceutics of China, Key Laboratory of Cardiovascular Research, Ministry of Education), College of Pharmacy, Harbin Medical University, Harbin 150081, Heilongjiang, China; 4 Department of Orthopedic Surgery, The Second Affiliated Hospital, Harbin Medical University, Harbin 150001, China

**Keywords:** small molecule drugs, long noncoding RNAs, network, pharmacological analysis, tissue-specificity

## Abstract

Long noncoding RNAs (lncRNAs) have multiple regulatory roles and are involved in many human diseases. A potential therapeutic strategy based on targeting lncRNAs was recently developed. To gain insight into the global relationship between small molecule drugs and their affected lncRNAs, we constructed a small molecule lncRNA network consisting of 1206 nodes (1033 drugs and 173 lncRNAs) and 4770 drug-lncRNA associations using LNCmap, which reannotated the microarray data from the Connectivity Map (CMap) database. Based on network biology, we found that the connected drug pairs tended to share the same targets, indications, and side effects. In addition, the connected drug pairs tended to have a similar structure. By inferring the functions of lncRNAs through their co-expressing mRNAs, we found that lncRNA functions related to the modular interface were associated with the mode of action or side effects of the corresponding connected drugs, suggesting that lncRNAs may directly/indirectly participate in specific biological processes after drug administration. Finally, we investigated the tissue-specificity of drug-affected lncRNAs and found that some kinds of drugs tended to have a broader influence (e.g. antineoplastic and immunomodulating drugs), whereas some tissue-specific lncRNAs (nervous system) tended to be affected by multiple types of drugs.

## INTRODUCTION

Increasing research has shown that long noncoding RNAs (lncRNAs) are pivotal regulators in many biological processes, as well as in the generation and progression of various diseases [1–3]. Many lncRNAs are aberrantly expressed in different pathological states [4], and the restoration of lncRNA expression has dramatic therapeutic potential on diseases, making it a novel therapeutic strategy [5].

Currently, pharmacotherapy is the most effective strategy in the treatment of some diseases. Small molecules are widely used due to their simple structure. In recent years, small molecules are found to either have therapeutic effects or induce side-effects/toxicity through the regulation of lncRNAs [6, 7]. Meanwhile, the differently expressed lncRNAs are also indicators of predicting drug sensitivity and resistance, especially in treatment of cancer [8, 9]. Therefore, a lncRNA-based therapeutic strategy hopefully could make personalized medicine become more realistic. However, structure-based approach such as molecular docking has been difficult to achieve as predicting the exact structure of lncRNAs remains a challenge. Another approach based on the transcriptional response might be appropriate to investigate the global relationships between small molecules (drugs) and lncRNAs.

Enormous array-based expression profile resources have promoted the development of research on small molecules. For example, Connectivity Map (CMap), which is a genome-wide transcriptional expression dataset of selected human cells treated with bioactive small molecules, pioneered the systemic transcriptional response-based approach [10]. Compared to the array-based expression profiles, there are not enough publicly available RNA-seq datasets of drug treatments yet, and this has been a limitation to the lncRNA-targeting therapy [11]. This limitation could be alleviated by a novel method called probe re-annotation in which microarray probes are reannotated for investigating lncRNA expression [12]. This approach has been successfully used for the functional annotation of lncRNAs in various studies [11]. We have reannotated the microarray data from the CMap database in our previous work named LNCmap, which successfully characterized the connections among diseases, lncRNAs and small molecules [13].

To investigate the global relationships between small molecules (drugs) and lncRNAs, we obtained the lncRNA expression profiles affected by small molecules from the LNCmap. We constructed a global small molecule lncRNA network (SMLN) in which nodes represented drugs or lncRNAs. Starting from the bipartite SMLN, we generated two biologically relevant network projections: the small molecule-small molecule network (SSN), in which nodes represented drugs, and two drugs were connected if they shared at least one lncRNA; and the lncRNA-lncRNA network (LLN), in which nodes were lncRNAs and two lncRNAs were connected if they significantly shared small molecules. Then we (i) investigated the pharmacological similarities of linked small molecule pairs in the SSN, (ii) explored the function of modules of the LLN in the responses to drug treatment, and (iii) analyzed the tissue specificity of lncRNAs after drug treatment.

## RESULTS

### Construction of a small molecule lncRNA network (SMLN)

**Figure 1:**
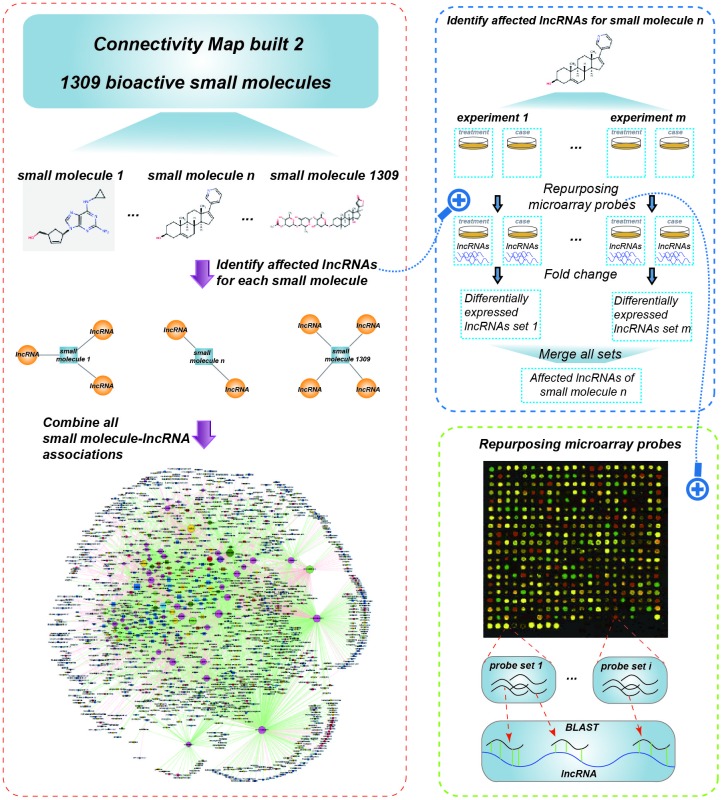
**Schematic data flowchart of SMLN.**

**Figure 2:**
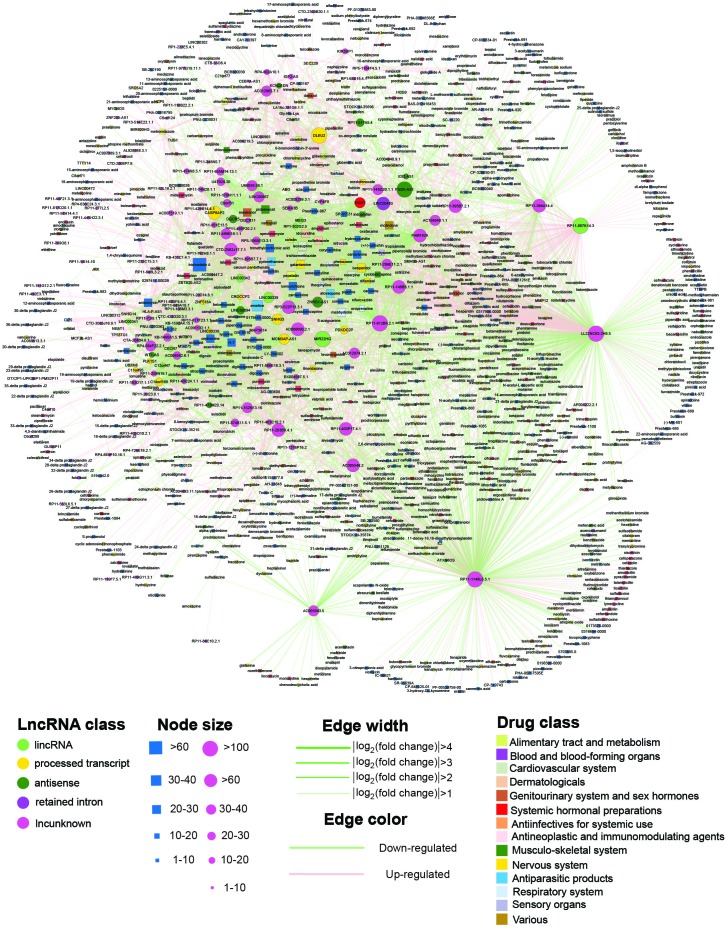
**The SMLN network.** The rectangles and circles in the network correspond to small molecules and lncRNAs, respectively. A small molecule and a lncRNA are connected by an edge if the lncRNA differentially expressed when treated with this small molecule. Colors represent different lncRNA and small molecule classes.

To construct the global SMLN, we retrieved lncRNA expression profiles affected by small molecules from LNCmap [13]. LNCmap reannotated the genome-wide transcriptional expression data from the CMap database, which contains 1,309 bioactive small molecules corresponding to 6100 instances (experiments). Among them, 5,916 microarray profiles were reannotated in LNCmap, including 237 lncRNA signatures and 1,262 small molecule drugs. We then used fold-change analysis to identify differentially expressed lncRNAs (DEL) for every instance with |log2fold change|>1 from the corresponding treatment and control microarray profiles. The DELs were merged if the corresponding experiments belonged to the same small molecules, and these lncRNAs were considered the affected lncRNAs for this small molecule (Figure 1). Then, we obtained a bipartite SMLN consisting of 1,005 small molecules and 173 lncRNAs (Figure 2 and Supplementary Dataset 1). We generated two biologically relevant network projections: the SSN and the LLN. If most small molecules specifically affected a single lncRNA, the LLN would consist of isolated nodes with few or no edges between them. Instead, the LLN displayed close connections between different lncRNAs. One reason for this phenomenon might be that there were over 1,000 small molecules compared with only 173 lncRNAs in the SMLN. The number of shared small molecules between different lncRNA pairs spanned a wide range. To improve the specificity of lncRNA pairs, we adopted a hypergeometric test to generate a more specific LLN (see Materials and Methods).

### The basic properties of the SMLN

The SMLN was composed of 1,206 nodes (1,033 small molecules and 173 lncRNAs) and 4,770 edges (2,628 downregulated and 2,142 upregulated) (Figure 2). All nodes were in one giant component, suggesting that the small molecules and lncRNAs were closely connected in the SMLN. The degree of distribution of the small molecule and lncRNA nodes followed power law distributions with a slope of -0.947 and -0.850, respectively, and R^2^=0.874 and 0.532, respectively (Supplementary Figure 1A, 1B and Supplementary Dataset 2). Thus, the SMLN was scale-free [14].

The degree of small molecule nodes spanned a wide range from 1 to 87. The highest degree node was trichostatin A (TSA), an organic compound that serves as an antifungal antibiotic and selectively inhibits class I and II mammalian histone deacetylases (HDACs) [15]. TSA can broadly alter gene expression by interfering with the removal of acetyl groups from histones [16, 17]. It is also a member of a larger class of histone deacetylase inhibitors that have a broad spectrum of epigenetic activities [16, 17]. The second highest degree small molecule node (degree=46) was emetine, an anti-malaria drug that was recently found to have broad anticancer activity in many types of malignancies including breast, colon, prostate, skin, and lymphoid tumors by inhibiting NF-κB signaling or regulating the RNA splicing of members of the Bcl-2 family [18, 19]. Although there are no specific reports about emetine and lncRNAs, it was linked to many lncRNAs, partly because of its broad anticancer effects. Interestingly, we found that other highly connected nodes, namely anisomycin and idoxuridine (degree: 39 and 38, respectively) could inhibit protein/DNA synthesis. Anisomycin is a potent apoptosis inducer that functions by activating JNK/SAPK and inhibiting protein/DNA synthesis during translation [20, 21]. Idoxuridine, which is used as an antiviral agent, is an analog of deoxyuridine, an inhibitor of viral DNA synthesis [22]. The high connectivity may have been due to their activity related to apoptosis and the inhibition of protein/DNA synthesis.

**Figure 3:**
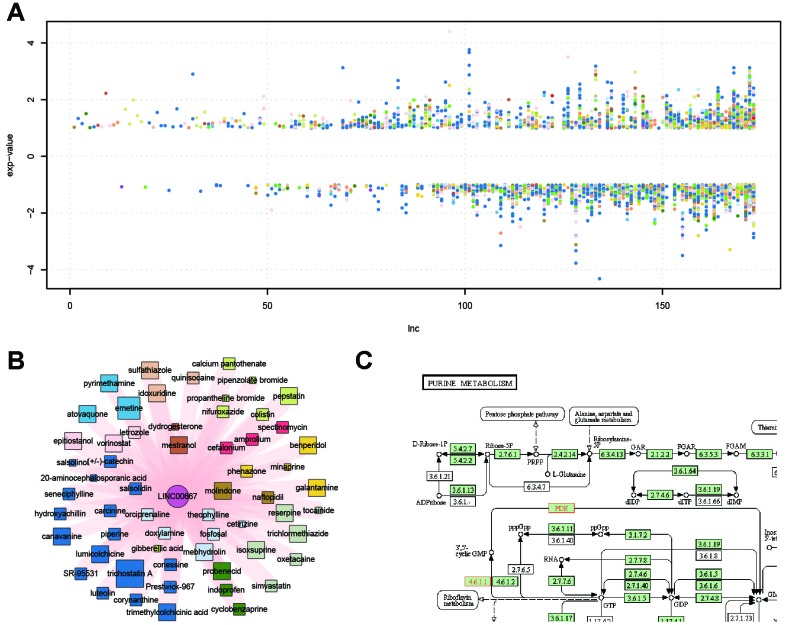
**LncRNA expression values and functional characteristics.** (**A**) Fold change value of lncRNAs affected by drugs; colors represent different ATC codes of drugs affected by the specific lncRNA. (**B**) Sub-network of LINC00667 and the related drugs: LINC00667 was always up-regulated after drug treatment. (**C**) Functional characteristics of LINC00667 by pathway enrichment with its co-expressed protein-coding genes.

Similar to the small molecule nodes, the lncRNA nodes also displayed evident differences in connection (range, 1–366). The lncRNA node with the highest degree was RP11-1148L6.5.1. There are no functional studies about this lncRNA. To date, few lncRNAs have been functionally annotated. Of seven lncRNAs with a degree >100, only *DLEU2* (Deleted in Lymphocytic leukemia 2) is well studied. It encodes a pair of critical pro-apoptotic microRNAs, miR-15a/16-1, which are critical for the increased survival exhibited by chronic lymphocytic leukemia cells [23]. Chen et al. indicated that the HDAC inhibitor TSA, the most-connected small molecule in the SMLN, could upregulate the expression of miR-15a/16-1, residing in the host tumor suppressor *DLEU2* gene [24]. Furthermore, in our SMLN, TSA could also upregulate *DLEU2* (log2 fold change = 1.4), suggesting that our SMLN could identify a promising cancer therapy via targeting lncRNAs [23]. In addition, we showed that the fold change value varied within a wide range (Figure 3A). All fold-change values of LINC00667 were positive, indicating that the expression of this lncRNA is always upregulated in response to drug treatment (Figure 3B). The function of this lncRNA has not been well-studied. Thus, pathway enrichment analysis was used to examine the function of LINC00667. The results showed that it was related to purine metabolism, which shows clear relevance to various cancers such as bladder cancer, kidney cancer and prostate cancer (Figure 3C) [25].

### Pharmacological similarity of small molecule pairs

In our SMLN, the connections between small molecules and lncRNAs revealed the non-coding transcriptional responses after drug treatment. Studies have indicated that drugs leading to similar transcriptional responses tend to have similar pharmacological properties [10, 26]. Thus, we first constructed a SSN in which nodes represent drugs, and two small molecules are connected if they share at least one lncRNA (Supplementary Figure 2 and Supplementary Dataset 3). Then, we investigated whether the connected small molecules (drugs) tend to have similar pharmacological properties.

**Figure 4:**
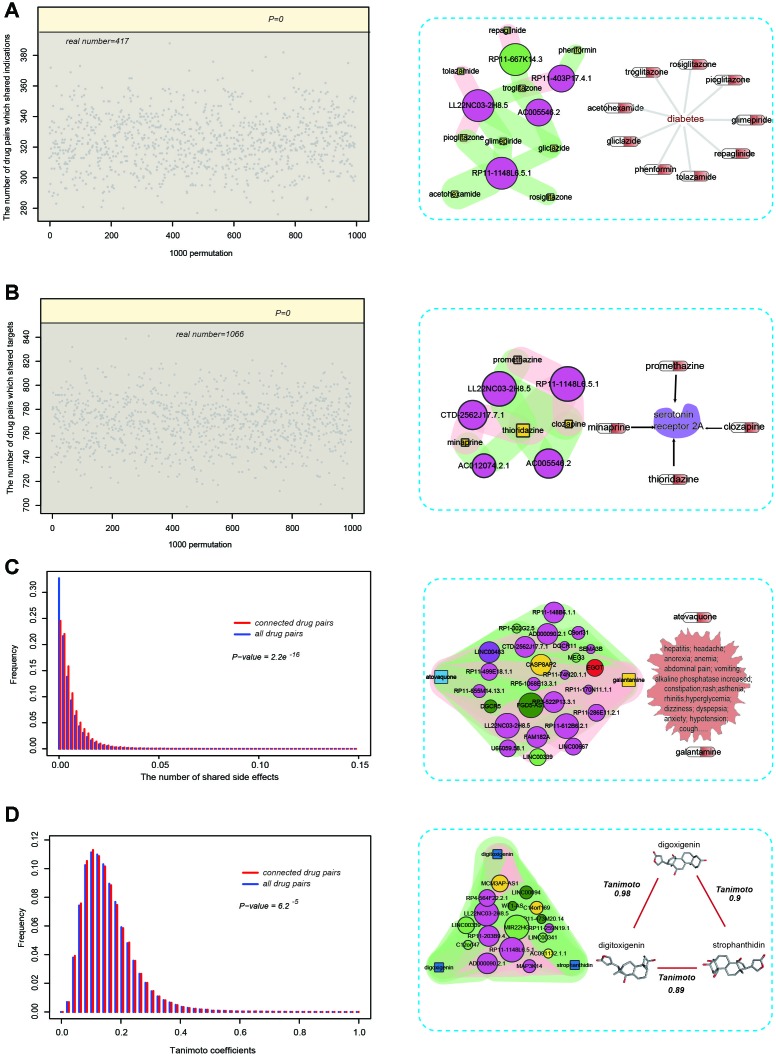
**Pharmacological properties of connected drug pairs in the SSN.** (**A**, left) 417 drug pairs with the same lncRNAs shared the same indications, compared with 1000 permutations. (**A**, right) Acetohexamide and gliclazide were connected to the same lncRNAs and they were all used for the treatment of diabetes. (**B**, left) 1066 drug pairs with the same lncRNAs shared the same drug targets, compared with 1000 permutations. (**B**, right) Minaprine and thioridazine shared the same lncRNA and both target the serotonin receptor 2A (HTR2A). (**C**, left) The proportion of shared side effects by drug pairs with the same lncRNAs (red), compared with the proportion of shared side effects among the total drug pairs in the SIDER database (blue). (**C**, right) Atovaquone and galantamine shared the same lncRNAs, although they belong to different categories, and could cause many of the same side effects. (**D**) Drug pairs with the same lncRNAs had higher TC scores.

We examined a total of four properties of connected drug pairs in SSN: indications, targets, side effects, and 2-D structural similarity (Figure 4). Firstly, we investigated whether connected drug pairs tend to share the same indications (treat the same disease). For this purpose, we first downloaded the dataset of drug–indication association from the report published by Yildirim et al. (see Materials and Methods) [27]. Of these connected drug pairs, 417 shared the same indications. We then generated randomized drug pairs 1,000 times. We found that in the 1,000 random times, the number of randomized drug pairs sharing the same indications were <417, suggesting that connected pairs tended to share the same indications (P = 0) (Figure 4A, left). Some drugs such as acetohexamide and gliclazide were connected to the same lncRNAs, and they were all used for the treatment of diabetes (Figure 4A, right). Based on this result, we questioned whether connected drug pairs tend to share the same targets. We found that some connected drugs such as minaprine and thioridazine both target serotonin receptor 2A (HTR2A), a protein associated with the susceptibility to schizophrenia and obsessive-compulsive disorder (Figure 4B, right) [28]. To further test this, we downloaded the drug-target association from the DrugBank database [29]. In the SMLN, 1,066 unique connected drug pairs targeted the same proteins. Similar to the aforementioned indications, we generated randomized drug pairs 1,000 times and there were no instances in which the number of randomized drug pairs sharing the same targets were more than 1,066, suggesting that connected drug pairs tended to share the same targets (Figure 4B, left). Then, we downloaded the public and accurate side-effect records from the side effect resource (SIDER), including 997 drugs corresponding to 4,492 side effects [30]. In the SMLN, there were 303 drugs recorded in the SIDER database. In the SIDER database, however, some side effects, such as dizziness and nausea, were caused by most drugs. To improve the specificity of similarity, we calculated the number of side effects shared by drug pairs rather than the number of drug pairs sharing the same side effects [26]. We found that the ratio of side effects shared by connected drug pairs was significantly higher than the number of side effects shared by other drug pairs in the SIDER database (P = 2.2e^−16^, Wilcoxon rank-sum test; Figure 4C, left), suggesting that two drugs connected by the same lncRNAs tended to cause the same side effects. Unlike the indications and targets, some connected drug pairs (atovaquone and galantamine), despite belonging to different categories, could cause the same side effects (Figure 4C, right), indicating that the non-coding transcriptional response might also capture such "heterogeneous" similarity.

Previously, a study indicated that pharmacologically similar drugs tend to have a similar structure [27]. Thus, we tested whether lncRNA-connected drugs tend to have a similar structure. For this purpose, we downloaded the SMILES files of small molecules in SSN and calculated the Tanimoto Coefficients (TC) of connected drug pairs and other small molecule pairs. We found that the TC scores of connected drug pairs were significantly higher than those of other small molecule pairs (P = 6.2e^−5^), Wilcoxon rank-sum test; Figure 4D, left), suggesting that the connected drugs tend to have a similar structure. Some connected small molecules pairs showing high structural similarity are shown in the right part of Figure 4D.

### Functional interface of drug-induced lncRNA modules

Similar to the SSN, we generated another biologically relevant network projection, namely the LLN, in which two lncRNAs are connected if they share significant numbers of small molecules (Supplementary Figure 3 and Supplementary Dataset 4). The connected lncRNA pairs affected by the same small molecules in the LLN might tend to have similar functions. Thus, the lncRNAs in one community of the LLN were considered to function "synergistically" because they were affected by the same or similar small molecules. We further proposed that lncRNAs, as members of more than one community, were more important and may be involved in key pathways related to therapeutic effects or the indication of corresponding drugs, because lncRNAs in multiple communities could be considered to be at the "interface" of different biological processes. Here, we investigated the functions of such interface lncRNAs and the relations to their connected drugs.

**Figure 5:**
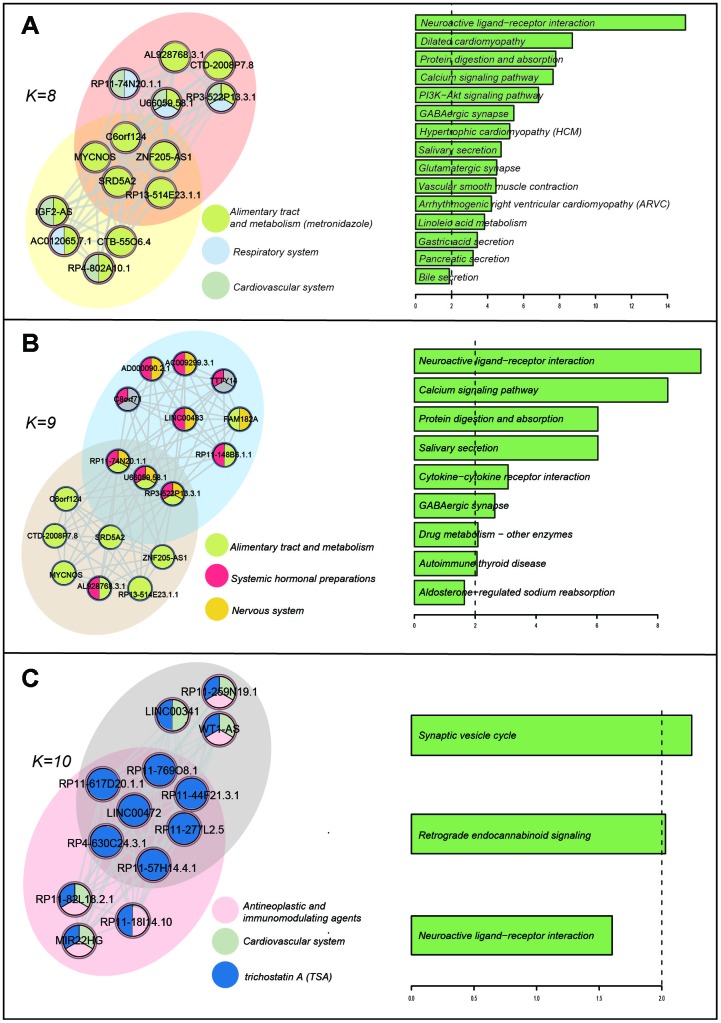
**Drug-induced lncRNA modules and enriched KEGG pathways.** (**A**) k=8. (**B**) k=9. (**C**) k=10. The K represents the number of the nodes in the modules.

First, we inferred the functions of lncRNAs through their co-expressing mRNAs across all the re-annotated microarrays in the CMap according to the "Guilt By Association" principle [12]. We then defined a lncRNA-lncRNA module as a clique, which is a maximal complete subgraph using Cfinder [31]. Each module had a unique composition of lncRNAs, allowing the same lncRNAs or the same pairs to occur in more than one module. Here, we adopted K=8, 9, and 10, because a low k-value generated numbers of extensive and less tightly connected lncRNA modules, leading to a high degree of overlap [14]. We displayed the modules of the LLN and the interface between them when the k-value was 8, 9, and 10 (the left part of Figure 5). The lncRNAs are colored according to the majority of their connected drug ATC classes. The right part of Figure 5 shows the significantly enriched pathways of co-expressing mRNAs of the "interface" lncRNAs. When the k-value was 8, there were two communities and 5 lncRNAs located at the interface of two modules (Figure 5A). The interface lncRNAs were mainly affected by drugs of "Alimentary tract and metabolism", and these lncRNAs were involved in key pathways such as the calcium signaling pathway, the PI3K-Akt signaling pathway, and the GABAergic synapse. We found that these pathways (such as "Gastric acid secretion" and "Pancreatic secretion") were not only related to "Alimentary tract and metabolism" drugs, but also related to the other two kinds of drugs ("Respiratory system" and "Cardiovascular system"). For example, cardiovascular disease pathways such as "Dilated cardiomyopathy" and "Hypertrophic cardiomyopathy" were identified. Furthermore, some key pathways such as the "PI3K-Akt signaling pathway" have comprehensive biological roles and are involved in the indications of these kinds of drugs [32, 33].

When the k-value was 9, two communities had three interface lncRNAs. We also found that these interface lncRNAs were involved in key pathways related to the drugs of "Alimentary tract and metabolism", "Systemic hormonal preparations", and "Nervous system"(Figure 5B). For example, the pathway "Cytokine-cytokine receptor interaction" plays a crucial role as intercellular regulator and mobilizer of cells engaged in innate as well as adaptive inflammatory host defenses, cell growth, differentiation, cell death, angiogenesis, and development and repair processes [34], and it is also related to many indications of the above three kinds of drugs. Another pathway, "Drug metabolism-other enzymes", was also detected. We found that this pathway was not only related to drug metabolism, but also contained enzymes encoded by genes related to metabolic diseases (i.e. xanthinuria and hyperbilirubinemia). When the k-value was 10, there were five interface lncRNAs that were all affected by the drug TSA. We found that the functions of these lncRNAs focused on the nervous system, and TSA had some side effects affecting the nervous system (Figure 5C). These results indicated that these interface lncRNAs may be directly/indirectly affected by drugs and broadly participated in the process of drug therapeutics, side effects, and metabolism.

### Tissue-specificity of drug-affected lncRNAs

Previous studies indicated that lncRNA expression may be limited to selected tissues or subpopulations of cells [35, 36]. The exclusively tissue-specific expression pattern of lncRNAs provides a unique opportunity for specific regulation by lncRNA-targeting therapeutics [35, 36]. In our study, lncRNAs in the SMLN were induced by different drugs, which were classified according to ATC (Anatomical Therapeutic Chemical Classification System) categories. This raises the issue of whether drugs tend to affect the lncRNAs expressed in their corresponding target tissues.

**Figure 6:**
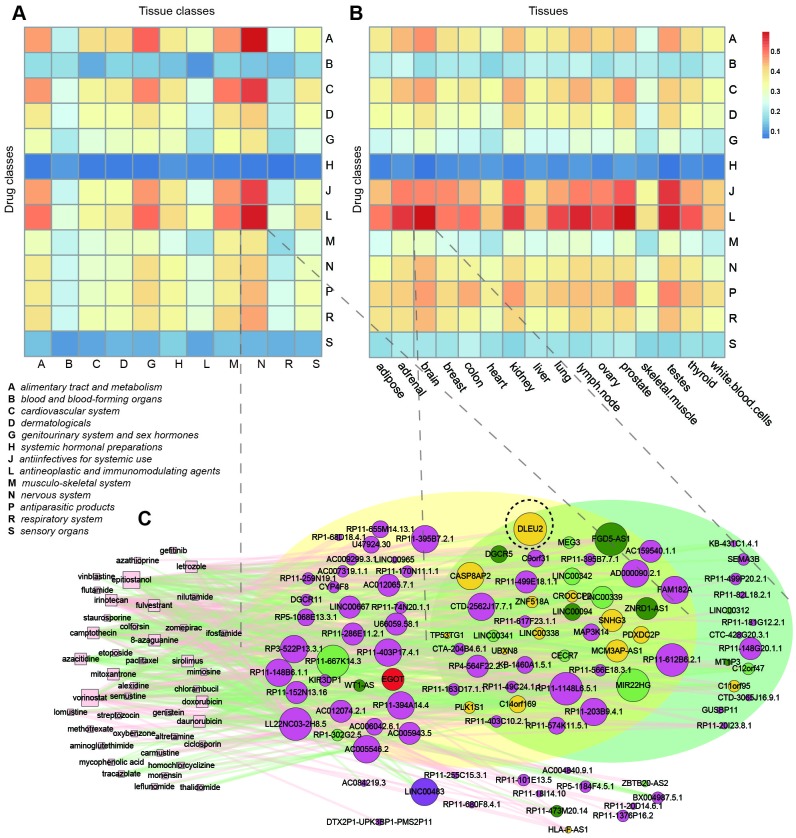
**Tissue-specificity of drug-affected lncRNAs.** (**A**) Jaccard coefficients of lncRNAs between 13 drug classes and tissues of 11 anatomical classes. (**B**) Jaccard coefficients of lncRNAs between 13 drug classes and 16 tissues. (C) Sub-network of the SMLN with drugs belonging to the (L) code and their affected lncRNAs.

To obtain a global overview of the anatomical distribution of the lncRNAs affected by different drug classes, we first reannotated the microarray dataset of GSE1133 obtained for 176 lncRNAs across 79 healthy tissues, and identified tissue-specific lncRNAs for each tissue (Supplementary Table 1) [37]. Then, we classified these 79 tissues into 11 anatomical classes according to ATC categories. We calculated the Jaccard coefficient of numbers of overlapped lncRNAs between 13 drug classes and tissues of 11 anatomical classes (Figure 6A). We found that drugs from all drug classes do not affect lncRNAs expressed in the corresponding anatomical classifications. Instead, some drugs tended to affect broadly tissue-specific lncRNAs. For example, anti-infectives (J) and antineoplastic and immunomodulating (L) drugs were more spread than other kinds of drugs. This might be due to the systemic therapeutic use or side effects of these two kinds of drugs. Certain types of drugs and tissues had obviously higher Jaccard coefficients [for example, (L) drugs and nervous system; (**C**) drugs (cardiovascular system) and nervous system]. On the other hand, we found that some tissue-specific lncRNAs (for example, nervous system specific lncRNAs) were prone to be affected by many kinds of drugs. The highest Jaccard coefficient was found between alimentary tract and metabolism drugs and the nervous system, suggesting that this kind of drug might predominantly affect nervous system-specific lncRNAs. To ensure the robustness of our results, we also calculated the Jaccard coefficients of overlapped lncRNAs between 13 drug classes and 16 tissues by processing the RNA-seq data of 16 normal human individual tissues from the ArrayExpress database (ERP000546) [38] (see Materials and Methods). Although the tissues were different from those of GSE1133, some similar results were observed (Figure 6B). For example, drugs of (J) and (L) codes were more spread; furthermore, higher Jaccard coefficients were also observed in (L) drugs and the nervous system (brain).

To better investigate the mechanism of tissue-specificity of lncRNAs induced by drugs, we extracted the sub- network from the SMLN of drugs belonging to the (L) code and their affected lncRNAs (Figure 6C). Many lncRNAs were expressed in the nervous system of GSE1133 or in the brain of ERP000546. Some of the LncRNAs were related to cancers and expressed in the brain. For example, DLEU2 (black dotted circle), a potential therapeutic target of chronic lymphocytic leukemia [23], was expressed in the brain and associated with axon degeneration in the brain [39].

## DISCUSSION

LncRNAs have multiple biological function and are considered as potential therapeutic targets for diseases [40–42]. LncRNA-targeting therapeutics can modulate disease pathways that have previously been considered to be intractable [43]. However, the global relationships between drugs and their affected lncRNAs and the therapeutic potential of lncRNAs are not well-characterized. Currently, a common way to analyze lncRNA expression is RNA-seq. However, publicly available RNA-seq datasets of drug treatments are relatively limited compared with array-based expression profiles. Therefore, we extracted lncRNA expression profiles of drug treatments from LNCmap, which adopts a strict threshold in the reannotation process, and obtained similar expression values than those of RNA-seq data.

Then, we constructed a global SMLN consisting of 1206 nodes (1033 small molecules and 173 lncRNAs), and 4770 edges (2628 downregulated and 2142 upregulated). Based on the SMLN, we found that connected drug pairs (two drugs sharing the same lncRNAs) tended to have similar pharmacological properties. Especially, these connected drug pairs tended to have similar structure, indicating the possibility of structure-based combinations between drugs and lncRNAs in our SMLN. Because two drugs may bind to the same "structural motifs" in the corresponding lncRNA, future investigation of lncRNA structures may reveal critical functional RNA motifs of lncRNAs susceptible to small molecule targeting [44]. Furthermore, we investigated the functional interface between drugs and lncRNA modules. By inferring the functions of lncRNAs through their co-expressing mRNAs, we found that the functions of lncRNAs in the modular interface were related to the mode of action or side effects of the corresponding connected drugs, suggesting that lncRNAs may directly/indirectly participate in biological processes after drug administration. Finally, we investigated whether drugs tend to affect the lncRNAs expressed in their corresponding target-tissue. By integrating the tissue-specific lncRNAs and drug-affected lncRNAs, we found that drugs from all drug classes did not affect the lncRNAs expressed in the corresponding anatomical tissues. Some kinds of drugs tended to have a broader influence (e.g. antineoplastic and immunomodulating drugs), whereas some tissue-specific lncRNAs tended to be affected by multiple kinds of drugs. This might be due to the broad effects of some systemic drugs. For example, (L) drugs were used for systemic antineoplastic and immunomodulating therapy. Another reason might be that there were only 173 lncRNAs that were reannotated, leading to an insufficient drug-lncRNA map. Nevertheless, we obtained some interesting findings.

To confirm the validity of our results, we also constructed the SMLN with fold change = 1.5 and fold change = 3 respectively and repeated some of the analyses for the network. We found that the SMLN networks with different fold changes were robust (Supplementary Table 2). This result indicated that our method was robust. There were some limitations in our study. First, there were only 173 lncRNAs in the SMLN. This was due to the strict threshold in the reannotation process and the limited data on drug effects. The SMLN can be improved by integrating additional drug-affected expression profiles and RNA-seq data from different resources. Another limitation of our study was the incompleteness of the functional annotation of lncRNAs. This limitation will be alleviated, to a great extent, by the development of functional genome and RNAi research, as well as the integration of bioinformatics databases.

In summary, we have constructed and analyzed the SMLN that provided a comprehensive picture of global associations between drugs and their affected lncRNAs. A better understanding of the relation between small molecule agents and lncRNAs would not only promote the repositioning and rational clinical use of these agents but also provides new insights into lncRNA-targeting therapeutics.

## MATERIALS AND METHODS

### Construction of the SMLN

Drugs and affected lncRNAs were obtained from the LNCmap (http://bio-bigdata.hrbmu.edu.cn/LncMAP/). The LNCmap extracted drug-affected lncRNA expression profiles by reannotating the microarray data from the CMap database. According to the pipeline of ncFANs [12], the LNCmap developed a similar computational method to reannotate lncRNAs from expression microarray of coding genes. LNCmap reannotated 5,916 microarray profiles, with 674 instances from the Human Genome U133 Set (HG-U133A) platform and 5242 instances from the GeneChip HT Human Genome U133 Array Plate Set (HT_HG-U133A) platform. We then used the R package affy to compute expression values for all lncRNA expression profiles and obtained log2-fold change values to identify differentially expressed lncRNAs (DEL). The DELs were merged if the corresponding experiments belonged to the same drug. After the above steps, we obtained 4,770 small molecule-lncRNA relationships, including 1,005 small molecules and 173 lncRNAs, and constructed a bipartite small molecule lncRNA network (SMLN). SMLN can be visualized by Cytoscape 3.0 [45] (Figure 2).

### Generating the LLN

We generated the LLN in which lncRNAs represented nodes and two lncRNAs were connected if they shared significant numbers of small molecules. Because of the marked differences between the number of lncRNAs (173) and small molecules (1005), lncRNAs were connected to each other closely. To improve the specificity and identify the more significant lncRNA pairs, we adopted a hypergeometric test to generate the LLN.

$$p=1-\overset{r-1}{\underset{x=0}{\sum }}\frac{\left(\begin{array}{c} t\\ x \end{array})(\begin{array}{c} m-t\\ n-x \end{array}\right)}{\left(\begin{array}{c} m\\ n \end{array}\right)}$$

Here, we collected m total small molecules in the SMLN, for each two lncRNAs i and j, t was the number of small molecules affected by lncRNA i, and n was the number of small molecules affected by lncRNA j, of which r was overlapped small molecules of the two small molecule sets. After calculating the P-value, we adopted the FDR-corrected q-values to reduce the false positive discovery rate. Significant lncRNA pairs (P<0.01, q-values<0.01) were obtained to construct the LLN.

### Datasets of pharmacological properties

### Indications

We collected the drug-indication associations from the study of Yildirim et al [27]. We also downloaded the drug-indication associations from Therapeutic Target Database (TTD) [46], then integrated the two datasets manually.

### Drug targets

We downloaded the drug-target associations from the DrugBank database [29], which is a unique bioinformatics and cheminformatics resource that combines detailed drug data with comprehensive drug target information. We obtained 399 small molecules in our SMLN.

### Side effects

We downloaded the drug side effect dataset from a public computer-readable resource, SIDER, which is a freely available database that contains information on marketed medicines and their recorded adverse drug reactions [30]. We collected 997 drugs corresponding to 4492 side effects, including 303 small molecules in the SMLN.

### Drug chemical similarity

We downloaded the SMILES files of small molecules in the SSN from the DrugBank database and Kyoto Encyclopedia of Genes and Genomes (KEGG, http://www.kegg.jp/kegg/drug/). We computed the TC scores of drug pairs using the Chemical Development Kit with default parameters [47].

### Pathway enrichment

Pathway enrichment analysis was implemented based on co-expressed protein-coding genes of lncRNAs by using SubpathwayMiner tools [48]. We calculated the Pearson’s correlation coefficient (PCC) between all reannotated lncRNA expression files and mRNA expression profiles of CMap. Using the setting |PCC|>0.5 and p < 0.01, we obtained the correlating mRNAs for pathway enrichment. The pathway enrichment was implemented by SubpathwayMiner with default parameters.

### Tissue-specificity

We used the GSE1133 dataset and the ArrayExpress database (ERP000546) to study the tissue-specificity of drug-affected lncRNAs. We first reannotated the microarray dataset of GSE1133 and obtained 176 lncRNAs across 79 healthy tissues; then, we calculated tissue specificity scores for lncRNAs and identified tissue-specific lncRNAs (score >0.8) for each tissue [49]. According to the ATC classification of tissues and drugs, tissue-specific lncRNAs and drug-affected lncRNAs were allocated to the ATC classification separately, and we calculated the Jaccard coefficient between the tissue ATC classification and drug ATC classification to measure the similarity between lncRNAs related to different classifications of tissue and drug. We used the ArrayExpress database (ERP000546) to calculate the Jaccard coefficients of lncRNAs between 13 drug classes and 16 tissues by processing the RNA-seq data of 16 normal human individual tissues.

## Supplementary Material

Supplementary Materials

Supplementary Figures

Supplementary Tables

Supplementary Dataset 1

Supplementary Dataset 2

Supplementary Dataset 3

Supplementary Dataset 4
