# Supplementary material for: System level characterization of small molecule drugs and their affected long noncoding RNAs
Source: Aging (Albany NY). 2019 Dec 18;11(24):12428–51. doi: 10.18632/aging.102581 (PMC6949102; doi:10.18632/aging.102581)
Supplement: Supplementary Materials [file aging-11-102581-s005..pdf]

SUPPLEMENTARY MATERIALS

MATERIALS AND METHODS

Construction of the SMLN

Drugs and affected lncRNAs were obtained from the LNCmap. The LNCmap extracted drug-affected lncRNA expression profiles by reannotating the microarray data from the CMap database. According to the pipeline of ncFANs [1], the LNCmap developed a similar computational method to reannotate lncRNAs from expression microarray of coding genes. LNCmap reannotated 5916 microarray profiles, with 674 instances from the Human Genome U133 Set (HG-U133A) platform and 5242 instances from the GeneChip HT Human Genome U133 Array Plate Set (HT_HG-U133A) platform. We then used the R package affy to compute expression values for all lncRNA expression profiles and obtained log2-fold change values to identify differentially expressed lncRNAs (DEL). The DELs were merged if the corresponding experiments belonged to the same drug. After the above steps, we obtained 4770 small molecule-lncRNA relationships, including 1005 small molecules and 173 lncRNAs, and constructed a bipartite small-molecule lncRNA network (SMLN).

Generating the LLN

We generated the LLN in which lncRNAs represented nodes and two lncRNAs were connected if they shared significant numbers of small molecules. Because of the marked differences between the number of lncRNAs (173) and small molecules (1005), lncRNAs were connected to each other closely. To improve the specificity and identify the more significant lncRNA pairs, we adopted a hypergeometric test to generate the LLN.

$$p = 1 - \sum_{x=0}^{r-1} \frac{\binom{t}{x}\binom{m-t}{n-x}}{\binom{m}{n}}$$

Here, we collected m total small molecules in the SMLN, for each two lncRNAs i and j, t was the number of small molecules affected by lncRNA i, and n was the number of small molecules affected by lncRNA j, of which r was overlapped small molecules of the two small-molecule sets. After calculating the P-value, we adopted the FDR-corrected q-values to reduce the false positive discovery rate. Significant lncRNA pairs ($P<0.01$, q-values<0.01) were obtained to construct the LLN.

Datasets of pharmacological properties

Indications

We collected the drug-indication associations from the study of Yildirim et al [2]. We also downloaded the drug-indication associations from Therapeutic Target Database (TTD) [3], then integrated the two datasets manually.

Drug targets

We downloaded the drug-target associations from the DrugBank database [4], which is a unique bioinformatics and cheminformatics resource that combines detailed drug data with comprehensive drug target information. We obtained 399 small molecules in our SMLN.

Side effects

We downloaded the drug side effect dataset from a public computer-readable resource, SIDER, which is a freely available database that contains information on marketed medicines and their recorded adverse drug reactions [5]. We collected 997 drugs corresponding to 4492 side effects, including 303 small molecules in the SMLN.

Drug chemical similarity

We downloaded the SMILES files of small molecules in the SSN from the DrugBank database and Kyoto Encyclopedia of Genes and Genomes (KEGG, http://www.kegg.jp/kegg/drug/). We computed the TC scores of drug pairs using the Chemical Development Kit with default parameters [6].

Pathway enrichment

Pathway enrichment analysis was implemented based on co-expressed protein-coding genes of lncRNAs by using SubpathwayMiner tools [7]. We calculated the Pearson correlation coefficient (PCC) between all reannotated lncRNA expression files and mRNA expression profiles of CMap. Using the setting $|PCC|>0.5$ and $p < 0.01$, we obtained the correlating mRNAs for pathway enrichment. The pathway enrichment was implemented by SubpathwayMiner with default parameters.

Tissue-specificity

We used the GSE1133 dataset and the ArrayExpress database (ERP000546) to study the tissue-specificity of

drug-affected lncRNAs. We firstly re-annotated the microarray dataset of GSE1133 and obtained 176 lncRNAs across 79 healthy tissues; then, we calculated tissue specificity scores for lncRNAs and identified tissue-specific lncRNAs (score >0.8) for each tissue [8]. According to the ATC classification of tissues and drugs, tissue-specific lncRNAs and drug-affected lncRNAs were allocated to the ATC classification separately, and we calculated the Jaccard coefficient between the tissue ATC classification and drug ATC classification to measure the similarity between lncRNAs related to different classifications of tissue and drug. We used the ArrayExpress database (ERP000546) to calculate the Jaccard coefficients of lncRNAs between 13 drug classes and 16 tissues by processing the RNA-seq data of 16 normal human individual tissues.

The basic properties of the SMLN

The degree of small-molecule nodes spanned a wide range from 1 to 87. The highest degree node was trichostatin A (TSA), an organic compound that serves as an antifungal antibiotic and selectively inhibits class I and II mammalian histone deacetylases (HDACs) [9]. TSA can broadly alter gene expression by interfering with the removal of acetyl groups from histones [10, 11]. It is also a member of a larger class of histone deacetylase inhibitors that have a broad spectrum of epigenetic activities [10, 11]. The second highest degree small molecule node (degree=46) was emetine, an anti-malaria drug that was recently found to have broad anticancer activity in many types of malignancies including breast, colon, prostate, skin, and lymphoid tumors by inhibiting NF-κB signaling or regulating the RNA splicing of members of the Bcl-2 family [12, 13]. Although there are no specific reports about emetine and lncRNAs, it was linked to many lncRNAs, partly because of its broad anticancer effects. Interestingly, we found that other highly-connected nodes, namely anisomycin and idoxuridine (degree: 39 and 38, respectively) could inhibit protein/DNA synthesis. Anisomycin is a potent apoptosis inducer that functions by activating JNK/SAPK and inhibiting protein/DNA synthesis during translation [14, 15]. Idoxuridine, which is used as an antiviral agent, is an analog of deoxyuridine, an inhibitor of viral DNA synthesis [16]. The high connectivity may have been due to their activity related to apoptosis and the inhibition of protein/DNA synthesis.

Similar to the small molecule nodes, the lncRNA nodes also displayed evident differences in connection (range, 1–366). The lncRNA node with the highest degree was RP11-1148L6.5.1. There are no functional studies about this lncRNA. To date, few lncRNAs have been functionally annotated. Of seven lncRNAs with a degree >100, only DLEU2 (Deleted in Lymphocytic lEUkemia 2) is well studied. It encodes a pair of critical pro-apoptotic microRNAs, miR-15a/16-1, which are critical for the increased survival exhibited by chronic lymphocytic leukemia cells [17]. Chen et Al. indicated that the HDAC inhibitor TSA, the most-connected small molecule in the SMLN, could upregulate the expression of miR-15a/16-1, residing in the host tumor suppressor *DLEU2* gene [18]. Furthermore, in our SMLN, TSA could also upregulate DLEU2 (log2 fold change = 1.4), suggesting that our SMLN could identify a promising cancer therapy via targeting lncRNAs [17].

REFERENCES

1. Liao Q, Liu C, Yuan X, Kang S, Miao R, Xiao H, Zhao G, Luo H, Bu D, Zhao H, Skogerbø G, Wu Z, Zhao Y. Large-scale prediction of long non-coding RNA functions in a coding-non-coding gene co-expression network. Nucleic Acids Res. 2011; 39:3864–78. https://doi.org/10.1093/nar/gkq1348 PMID:21247874

2. Yildirim MA, Goh KI, Cusick ME, Barabási AL, Vidal M. Drug-target network. Nat Biotechnol. 2007; 25:1119–26. https://doi.org/10.1038/nbt1338 PMID:17921997

3. Li YH, Yu CY, Li XX, Zhang P, Tang J, Yang Q, Fu T, Zhang X, Cui X, Tu G, Zhang Y, Li S, Yang F, et al. Therapeutic target database update 2018: enriched resource for facilitating bench-to-clinic research of targeted therapeutics. Nucleic Acids Res. 2018; 46:D1121–D1127. https://doi.org/10.1093/nar/gkx1076 PMID:29140520

4. Law V, Knox C, Djoumbou Y, Jewison T, Guo AC, Liu Y, Maciejewski A, Arndt D, Wilson M, Neveu V, Tang A, Gabriel G, Ly C, et al. DrugBank 4.0: shedding new light on drug metabolism. Nucleic Acids Res. 2014; 42:D1091–97. https://doi.org/10.1093/nar/gkt1068 PMID:24203711

5. Kuhn M, Campillos M, Letunic I, Jensen LJ, Bork P. A side effect resource to capture phenotypic effects of drugs. Mol Syst Biol. 2010; 6:343. https://doi.org/10.1038/msb.2009.98 PMID:20087340

6. Steinbeck C, Hoppe C, Kuhn S, Floris M, Guha R, Willighagen EL. Recent developments of the chemistry development kit (CDK) - an open-source java library for chemo- and bioinformatics. Curr Pharm Des. 2006; 12:2111–20. https://doi.org/10.2174/138161206777585274 PMID:16796559

7. Li C, Han J, Yao Q, Zou C, Xu Y, Zhang C, Shang D, Zhou L, Zou C, Sun Z, Li J, Zhang Y, Yang H, et al. Subpathway-GM: identification of metabolic subpathways via joint

power of interesting genes and metabolites and their topologies within pathways. Nucleic Acids Res. 2013; 41:e101. https://doi.org/10.1093/nar/gkt161 PMID:23482392

8. Liu MX, Chen X, Chen G, Cui QH, Yan GY. A computational framework to infer human disease-associated long noncoding RNAs. PLoS One. 2014; 9:e84408. https://doi.org/10.1371/journal.pone.0084408 PMID:24392133

9. Vanhaecke T, Papeleu P, Elaut G, Rogiers V. Trichostatin A-like hydroxamate histone deacetylase inhibitors as therapeutic agents: toxicological point of view. Curr Med Chem. 2004; 11:1629–43. https://doi.org/10.2174/0929867043365099 PMID:15180568

10. Drummond DC, Noble CO, Kirpotin DB, Guo Z, Scott GK, Benz CC. Clinical development of histone deacetylase inhibitors as anticancer agents. Annu Rev Pharmacol Toxicol. 2005; 45:495–528. https://doi.org/10.1146/annurev.pharmtox.45.120403.095825 PMID:15822187

11. Shankar S, Srivastava RK. Histone deacetylase inhibitors: mechanisms and clinical significance in cancer: HDAC inhibitor-induced apoptosis. Adv Exp Med Biol. 2008; 615:261–98. https://doi.org/10.1007/978-1-4020-6554-5_13 PMID:18437899

12. Miller SC, Huang R, Sakamuru S, Shukla SJ, Attene-Ramos MS, Shinn P, Van Leer D, Leister W, Austin CP, Xia M. Identification of known drugs that act as inhibitors of NF-kappaB signaling and their mechanism of action. Biochem Pharmacol. 2010; 79:1272–80. https://doi.org/10.1016/j.bcp.2009.12.021 PMID:20067776

13. Boon-Unge K, Yu Q, Zou T, Zhou A, Govitrapong P, Zhou J. Emetine regulates the alternative splicing of Bcl-x through a protein phosphatase 1-dependent mechanism. Chem Biol. 2007; 14:1386–92. https://doi.org/10.1016/j.chembiol.2007.11.004 PMID:18096507

14. Grollman AP. Inhibitors of protein biosynthesis. II. Mode of action of anisomycin. J Biol Chem. 1967; 242:3226–33. PMID:6027796

15. Hori T, Kondo T, Tabuchi Y, Takasaki I, Zhao QL, Kanamori M, Yasuda T, Kimura T. Molecular mechanism of apoptosis and gene expressions in human lymphoma U937 cells treated with anisomycin. Chem Biol Interact. 2008; 172:125–40. https://doi.org/10.1016/j.cbi.2007.12.003 PMID:18241849

16. Seth AK, Misra A, Umrigar D. Topical liposomal gel of idoxuridine for the treatment of herpes simplex: pharmaceutical and clinical implications. Pharm Dev Technol. 2004; 9:277–89. https://doi.org/10.1081/PDT-200031432 PMID:15458233

17. Kasar S, Underbayev C, Yuan Y, Hanlon M, Aly S, Khan H, Chang V, Batish M, Gavrilova T, Badiane F, Degheidy H, Marti G, Raveche E. Therapeutic implications of activation of the host gene (Dleu2) promoter for miR-15a/16-1 in chronic lymphocytic leukemia. Oncogene. 2014; 33:3307–15. https://doi.org/10.1038/onc.2013.291 PMID:23995789

18. Chen CQ, Chen CS, Chen JJ, Zhou LP, Xu HL, Jin WW, Wu JB, Gao SM. Histone deacetylases inhibitor trichostatin A increases the expression of Dleu2/miR-15a/16-1 via HDAC3 in non-small cell lung cancer. Mol Cell Biochem. 2013; 383:137–48. https://doi.org/10.1007/s11010-013-1762-z PMID:23867991
