# Supplementary material for: System level characterization of small molecule drugs and their affected long noncoding RNAs
Source: Aging (Albany NY). 2019 Dec 18;11(24):12428–51. doi: 10.18632/aging.102581 (PMC6949102; doi:10.18632/aging.102581)
Supplement: Supplementary Figures [file aging-11-102581-s006..pdf]

SUPPLEMENTARY FIGURES

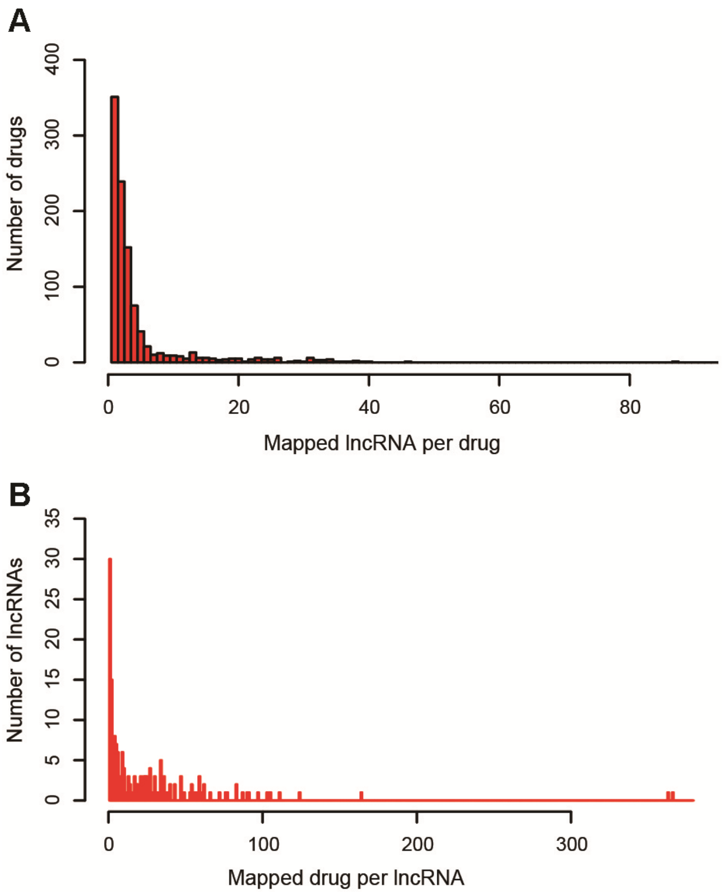

Supplementary Figure 1. The degree distribution of (**A**) lncRNAs and (**B**) drugs of the SMLN.

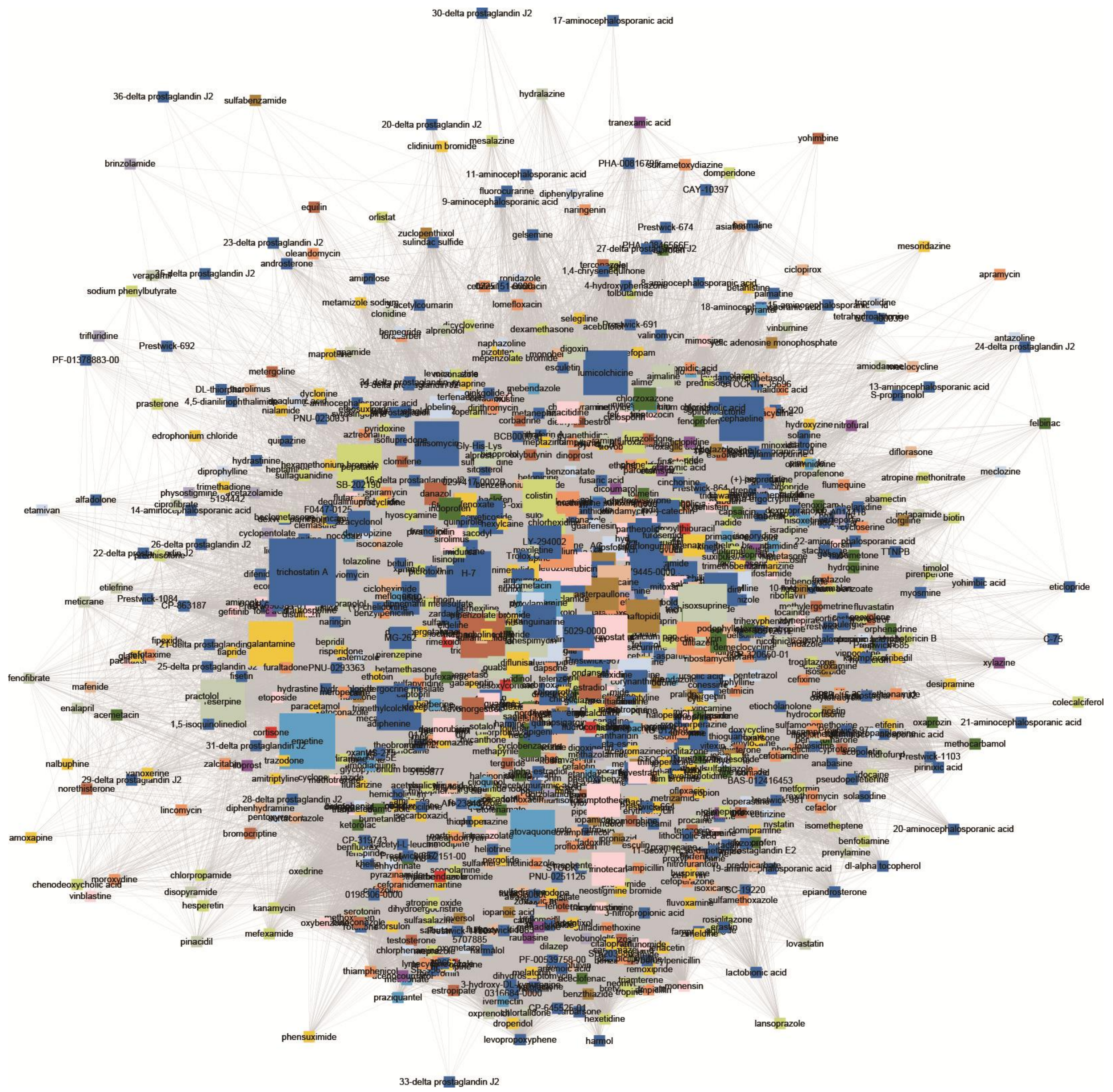

Supplementary Figure 2. The small molecule-small molecule network (SSN).

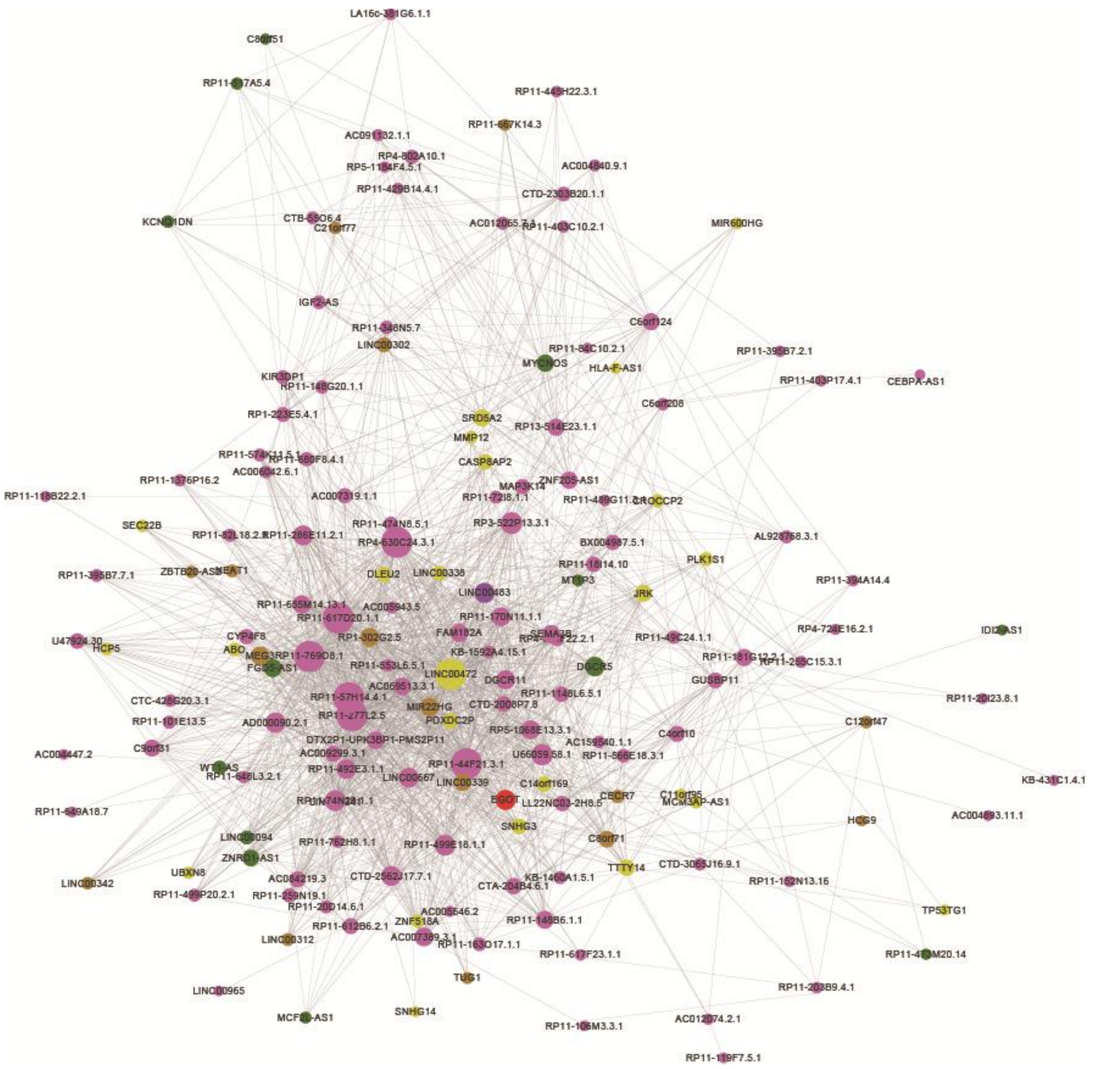

Supplementary Figure 3. The lncRNA-lncRNA network (LLN).
