# Supplementary material for: System level characterization of small molecule drugs and their affected long noncoding RNAs
Source: Aging (Albany NY). 2019 Dec 18;11(24):12428–51. doi: 10.18632/aging.102581 (PMC6949102; doi:10.18632/aging.102581)
Supplement: Supplementary Tables [file aging-11-102581-s004..pdf]

Supplementary Tables

Supplementary Table 1. Tissue-specific lncRNAs affected by drugs.

Tissue	lncRNAs
Brain Amygdala	ENSG00000269614, ENSG00000225465
subthalamic_nucleus	ENSG00000267670
TestiIntersitial	ENSG00000267163
DRG	ENSG00000267161, ENSG00000104725, ENSG00000179935, ENSG00000235280
ciliary_ganglion	ENSG00000266897, ENSG00000240291, ENSG00000232860, ENSG00000196696, ENSG00000230223, ENSG00000242125, ENSG00000237941, ENSG00000263072
fetal liver	ENSG00000261613
Pons	ENSG00000261496
TemporalLobe	ENSG00000261087
Uterus_Corpus	ENSG00000260339
Skeletal_Muscle_Psoas	ENSG00000254488, ENSG00000236772, ENSG00000263214, ENSG00000260588, ENSG00000259539, ENSG00000246777, ENSG00000249717, ENSG00000273032, ENSG00000235994, ENSG00000267934, ENSG00000232416, ENSG00000265242, ENSG00000241881, ENSG00000231690, ENSG00000197670, ENSG00000215765, ENSG00000176728
PB_CD19BCells	ENSG00000253701
cerebellum	ENSG00000253230
atrioventricular_node	ENSG00000249790, ENSG00000229645, ENSG00000218510, ENSG00000235947, ENSG00000227719, ENSG00000260532, ENSG00000005206, ENSG00000261071, ENSG00000266171, ENSG00000236234, ENSG00000249673
Superior_Cervical_Ganglion	ENSG00000246863, ENSG00000236901, ENSG00000270742, ENSG00000257310, ENSG00000241954, ENSG00000245532, ENSG00000182165, ENSG00000182873, ENSG00000269176, ENSG00000258442, ENSG00000255224, ENSG00000232710, ENSG00000236268, ENSG00000235725, ENSG00000225733, ENSG00000234912, ENSG00000225930, ENSG00000049319, ENSG00000259417, ENSG00000236673, ENSG00000237517, ENSG00000152268, ENSG00000251023, ENSG00000261460, ENSG00000260396, ENSG00000237697, ENSG00000231074, ENSG00000229921, ENSG00000272579, ENSG00000235072, ENSG00000224945, ENSG00000259291, ENSG00000226674, ENSG00000255443, ENSG00000233237, ENSG00000233864, ENSG00000228389, ENSG00000248175, ENSG00000249532, ENSG00000229589, ENSG00000227372, ENSG00000262179, ENSG00000186526, ENSG00000261097, ENSG00000204054, ENSG00000226803, ENSG00000248161, ENSG00000229582, ENSG00000110347, ENSG00000266830, ENSG00000233718, ENSG00000204623, ENSG00000260804, ENSG00000204625, ENSG00000031544, ENSG00000125804, ENSG00000260735, ENSG00000224063, ENSG00000272599, ENSG00000232274, ENSG00000164621, ENSG00000136315, ENSG00000006062, ENSG00000259322, ENSG00000187621, ENSG00000176075, ENSG00000260400, ENSG00000272216, ENSG00000186842, ENSG00000260743, ENSG00000215117, ENSG00000233791, ENSG00000231607, ENSG00000196756, ENSG00000249859, ENSG00000249267, ENSG00000234350, ENSG00000242687, ENSG00000259849, ENSG00000253116, ENSG00000232931, ENSG00000267659, ENSG00000241295, ENSG00000225670, ENSG00000272201, ENSG00000255318, ENSG00000183242, ENSG00000132832, ENSG00000241345, ENSG00000235733, ENSG00000176734, ENSG00000177853, ENSG00000204148, ENSG00000260619, ENSG00000144596, ENSG00000236256, ENSG00000228343, ENSG00000261646, ENSG00000273311, ENSG00000257303, ENSG00000215424, ENSG00000263753, ENSG00000235437, ENSG00000259758, ENSG00000131007, ENSG00000246263, ENSG00000266904, ENSG00000256185, ENSG00000260917, ENSG00000228463, ENSG00000237775, ENSG00000237438, ENSG00000263050, ENSG00000228350, ENSG00000249604, ENSG00000264727, ENSG00000259577
Uterus	ENSG00000237125
Testi_SeminiferousTubule	ENSG00000235824, ENSG00000088970
lymph node	ENSG00000228315
Appendix	ENSG00000226334
BM_CD71EarlyErythroid	ENSG00000215908
HEART	ENSG00000213994, ENSG00000269653
WHOLEBLOOD(JJV)	ENSG00000206337
Trigeminal_Ganglion	ENSG00000198221, ENSG00000270589, ENSG00000175873, ENSG00000266968, ENSG00000262420, ENSG00000225092, ENSG00000269318, ENSG00000251632, ENSG00000259644, ENSG00000227403, ENSG00000254389, ENSG00000237250, ENSG00000263198, ENSG00000259073, ENSG00000273487, ENSG00000258517, ENSG00000270074, ENSG00000231160, ENSG00000272711, ENSG00000167117,

	ENSG00000235865
Testi_GermCell	ENSG00000197210
Liver	ENSG00000188338
fetal brain	ENSG00000188070
AdrenalCortex	ENSG00000186594, ENSG00000214548
spinal cord	ENSG00000099869
PLACENTA	ENSG00000012171

Supplementary Table 2. Robustness of SMLN with Fold Change=2 and Fold Change=1.5.

Degree rank	Drug		lncRNA	
	FC=2	FC=1.5	FC=2	FC=1.5
1	trichostatin A	trichostatin A	RP11-1148L6.5.1	LL22NC03-2H8.5
2	emetine	trichlormethiazide	LL22NC03-2H8.5	RP11-1148L6.5.1
3	pepstatin	probenecid	RP11-612B6.2.1	RP11-667K14.3
4	anisomycin	0175029-0000	RP11-667K14.3	RP11-395B7.2.1
5	idoxuridine	trimethylcolchicinic acid	FGD5-AS1	RP11-612B6.2.1
6	lumicolchicine	emetine	MIR22HG	AC005546.2
7	probenecid	galantamine	DLEU2	FAM182A
8	reserpine	mestranol	FAM182A	AC159540.1.1
9	mestranol	pyrimethamine	RP11-403P17.4.1	AC012074.2.1
10	sulfathiazole	pepstatin	CTD-2562J17.7.1	DLEU2
11	molindone	quinisocaine	RP11-148B6.1.1	RP11-148G20.1.1
12	galantamine	benperidol	RP11-395B7.2.1	RP11-148B6.1.1
13	H-7	idoxuridine	AD000090.2.1	CTD-2562J17.7.1
14	pyrimethamine	reserpine	AC005546.2	RP11-403P17.4.1
15	trimethylcolchicinic acid	lumicolchicine	RP11-394A14.4	RP11-203B9.4.1
16	trichlormethiazide	metronidazole	RP3-522P13.3.1	RP11-394A14.4
17	lanatoside C	epitiostanol	RP11-203B9.4.1	FGD5-AS1
18	mebhydrolin	canavanine	CASP8AP2	RP11-1376P16.2
19	canavanine	sulfathiazole	LINC00483	BX004987.5.1
20	vorinostat	molindone	ZNRD1-AS1	AC012065.7.1
